# Microfluidic Organoids-on-a-Chip: Quantum Leap in Cancer Research

**DOI:** 10.3390/cancers13040737

**Published:** 2021-02-10

**Authors:** Fahriye Duzagac, Gloria Saorin, Lorenzo Memeo, Vincenzo Canzonieri, Flavio Rizzolio

**Affiliations:** 1 Department of Molecular Sciences and Nanosystems, Ca’ Foscari University of Venice, 30123 Venezia, Italy; fahriye.duzagac@unive.it (F.D.); gloria.saorin@unive.it (G.S.); 2 Department of Experimental Oncology, Mediterranean Institute of Oncology (IOM), 95029 Catania, Italy; lorenzo.memeo@grupposamed.com; 3 Pathology Unit, Centro di Riferimento Oncologico di Aviano (CRO) IRCCS, 33081 Aviano, Italy; vcanzonieri@cro.it; 4 Department of Medical, Surgical and Health Sciences, University of Trieste, 34127 Trieste, Italy

**Keywords:** organoids, tumoroids, microfluidics, organs-on-a-chip, living biobanks

## Abstract

**Simple Summary:**

Organoids, also known as self-organized 3D organ-like clusters, represent almost a physiological system for studying cells, stem cells, tissues, and diseases, especially cancer. Microfluidic organ-on-a-chip technology has gained beneficial interest in recent years due to its potential to be a pioneer in developing personalized treatments with a precise control of many parameters such as flow conditions and nutrient supply in a microscale. Further, the dynamic nature of such personalized systems also relies on the ability to easily combine the read-outs from blood samples, urine samples, biopsies, and primary tissues. This fast-evolving innovative technology precisely fit with the concept of precision medicine, which holds an infinite potential for personalized cancer treatments.

**Abstract:**

Organ-like cell clusters, so-called organoids, which exhibit self-organized and similar organ functionality as the tissue of origin, have provided a whole new level of bioinspiration for ex vivo systems. Microfluidic organoid or organs-on-a-chip platforms are a new group of micro-engineered promising models that recapitulate 3D tissue structure and physiology and combines several advantages of current in vivo and in vitro models. Microfluidics technology is used in numerous applications since it allows us to control and manipulate fluid flows with a high degree of accuracy. This system is an emerging tool for understanding disease development and progression, especially for personalized therapeutic strategies for cancer treatment, which provide well-grounded, cost-effective, powerful, fast, and reproducible results. In this review, we highlight how the organoid-on-a-chip models have improved the potential of efficiency and reproducibility of organoid cultures. More widely, we discuss current challenges and development on organoid culture systems together with microfluidic approaches and their limitations. Finally, we describe the recent progress and potential utilization in the organs-on-a-chip practice.

## 1. Introduction

Most of the current studies on cell and tissue regulation have relied on analyses of cells grown in a monolayer or suspension cell-culture models that fail to emulate the in vivo cellular micro-environment, therefore, lead to a rapid loss of function and de-differentiation [1]. Researchers have evolved the mammalian cells’ self-organizing capacity for a 3D cell culture system from primary tissues to avoid these limitations and aim to mimic the in vivo tissue microenvironment [2,3]. Organoid technology, an innovative three-dimensional (3D) model, has expeditiously increased in recent years and has become widespread by eliminating the gap between two-dimensional cultures and in vivo physiology. These self-organized organ-like-clusters could be derived from a variety of multipotent stem cells, such as embryonic stem cells (ESCs), induced pluripotent stem cells (iPSCs), adult stem cells, primary cells, as well as tumor cells in the 3D culture system and substantially mimic the organs from which they were derived, both in organization and in function.

## 2. Approaches for Organoid Cultures

**Figure 1:**
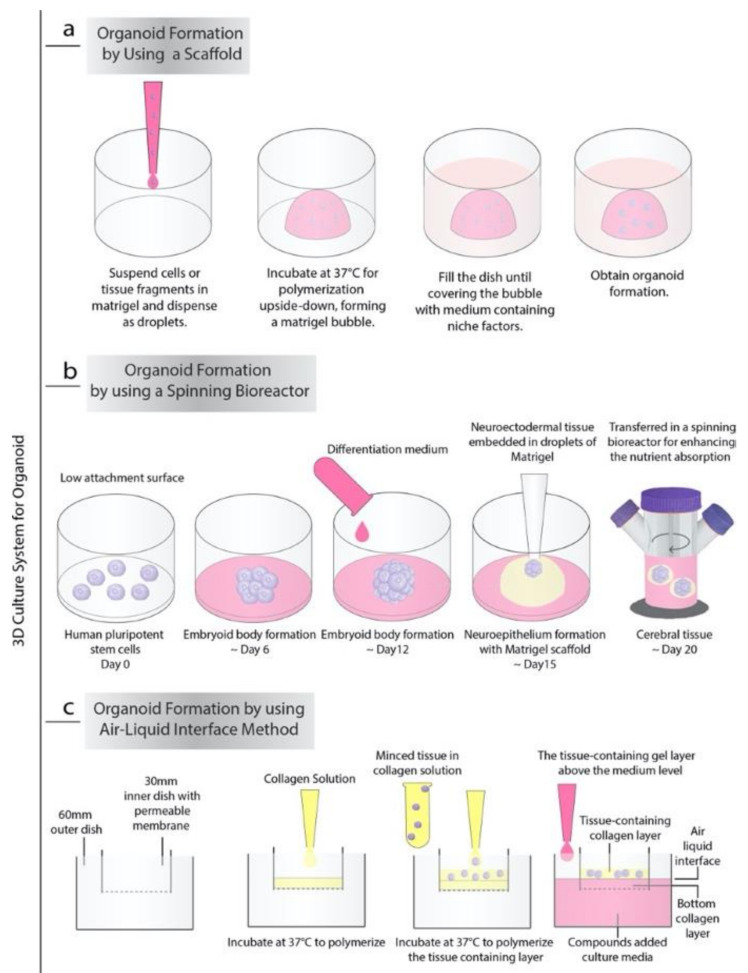
3D culture system of organoids. (**a**) Organoid formation using only a scaffold-Matrigel: (**b**) Organoid formation using spinning bioreactor: (**c**) Organoid formation using the Air-Liquid Interface method.

Currently, there are almost three general processes to produce organoids *in vitro*. The first very common and strikingly, the simplest of these approaches is based on extracellular matrix (ECM) (such as Matrigel or collagen-based) scaffolds (Figure 1a). In 2009, Sato T. et al. demonstrated that Lgr5+ stem cells isolated from the intestinal crypt or crypt itself have the ability to form intestinal crypt-villus units, which are self-regulating organoid structures without a non-epithelial cellular niche by using Matrigel supplemented with growth factors. These organoids were cultured for more than eight months without losing their characteristics, and microarray analysis showed that they were highly similar to freshly isolated intestinal crypts [4]. Sachs et al. showed that the embedding of proliferating organoids in a contracting floating collagen gel allows them to line up in macroscopic tubes to form a simple intestinal epithelium structure that contains all major types of small intestinal cells, including epithelium and stem cells [5]. The collagen-based matrix, including type 1 collagen, Ham’s F12 nutrient medium, and bicarbonate, showed similar results to a matrigel-based niche *in vitro*. Since most of the ingredients in matrigel are not fully defined comparing to collagen, it is hard to predict its effect on the human body. Additionally, given the matrigel-based niche’s ability to increase angiogenesis due to its ingredients such as cytokines and growth factors, for the in vivo organoid transplantation, the collagen base niche could be a valid alternative [6,7].

The second approach for generating organoids from the embryonic body (EB) or pluripotent stem cells is agitation using spinning-rotating bioreactors, which are often used to recapitulate human brain development in vitro (Figure 1b). As demonstrated by Lancaster et al., by providing the environment essential for intrinsic cues together with improved growth conditions, EBs generate neuroectodermal tissues [8]. Neuroectodermal tissues were embedded in Matrigel droplets to create the 3D scaffold structure to provide more inclusive tissue growth. Later, Matrigel droplets were transferred to a spinning bioreactor designed to increase nutrient absorption. The rapid development of brain tissue has been made by this method requiring only 8–10 days for the appearance of neural identity and 20–30 days to form specific brain regions. This method was also used to produce retinal organoids. In 2018, Di Stefano et al. reported an approach to culture retinal organoids from mouse pluripotent stem cells using the NASA-designed rotating-wall vessel (RWV) bioreactor [9]. With the RWV bioreactor, organoids successfully demonstrated well-defined morphology and advanced neuronal differentiation by forming ganglion and S-cone photoreceptors cells and increased proliferation.

The last method for generating organoids was the air-liquid interface (ALI) method (Figure 1c). The ALI method is mainly used to produce gastrointestinal organoids [10]. In this method, the top layer of the cells is exposed to the air while the basal surface is inside the liquid matrix medium produced by Matrigel, collagen, or other biological matrices. Researchers also combine the organotypic slice culture method with the ALI method and generate air-liquid interface cerebral organoids, which exhibit improved survival and morphology with extensive axon enlargements reminiscent of nerve tracts [11].

### 2.1. Niche Factors

So far, multiple types of healthy and cancer organoids have been efficiently established from several organs, including lung [12], gastric [13,14], colon [15,16], liver [17,18], pancreas [19], kidney [20], prostate [21], breast cancer [22], bladder [23], brain [24] and recently ovary [25,26,27,28]. Most advanced cancers show driver mutations and genomic alterations that allow the tumor to progress [29]. In that sense, selective culture conditions can be applied for most cancer organoids, considering the high penetrance of activating those driver mutations [30]. Cancer-specific organoid cultures require niche factors and ECM components modified from those already established with normal organoid cultures (Table 1). The culture system was created by supplementing growth factors that generate key endogenous niche signals besides using matrigel, collagen, or basement membrane extract (BME) as an ECM substitute.

#### 2.1.1. Niche Factors for Organoids that Derived from Mammary Epithelial Cells

Mammary epithelial cells are mainly grouped into two cell lineages, luminal and basal, based on their location within a bilayer of breast epithelium. The inner (luminal) cells secrete milk, while the contractile outer layer of myoepithelial (basal) cells ejects the milk. The Wnt signaling is an important pathway involved in different stages of breast development and oncogenesis due to its role in maintaining the self-renewal of progenitor cells and mammary gland stem cells [31]. 

Recent studies have shown that Rho-associated, coiled-coil containing protein kinase (ROCK) inhibitors increase colony formation in 3D culture and allow mammary epithelial cells to maintain regenerative capacity [32,33]. Additionally, compounds like forskolin, which stimulate adenylate cyclase and increase intracellular cyclic adenosine monophosphate (cAMP) levels, are commonly used for epithelial culture and promote polarization and lumen formation in the 3D culture system [33,34]. For the long-term culturing of mammary epithelial cells, ROCK inhibitor Y-27632 is required to form branching ducts with alveoli, and Wnt/R-spondin is crucial for their Wnt pathway activation effect. As an example, for breast cancer-specific organoids, the epithelial growth factor receptor (EGFR) ligand EGF to promote cell proliferation, the bone morphogenetic protein (BMP) inhibitor Noggin for cellular expansion, and LGR4/5 (leucine-rich repeat-containing G-protein-coupled receptor 4/5) ligand R-spondin, which acts as an agonist of the canonical Wnt/β-catenin signaling pathway are commonly used culture systems [22,35,36,37].

This so-called R-spondin culture system was also used for patient-derived lung-specific and prostate-specific cancer organoids with the addition of TGF-β pathway inhibitors, which help the maintenance of progenitor cells [21,38,39,40]. TGF- β pathway inhibitors followed by BMP/FGF stimulation are required due to their roles in generating a relatively unique population of progenitor cells that recapitulate the genuine development of lung and prostate [41]. 

#### 2.1.2. Niche Factors for Organoids that Derived from Gastrointestinal Tract

The gastrointestinal tract primarily develops from the endoderm, which derives from the epithelial tube as foregut, midgut, and hindgut. The foregut leads to the respiratory tract, stomach, pancreas, and liver, while the midgut forms the small intestine and ascending colon. The remaining part of the colon, large intestine, and rectum originate from the hindgut [42]. The separation of these three domains occurs by combining the growth factors with anteriorizing or posteriorizing effects [43]. The addition of BMP inhibitors, FGF, and Wnt activators in the embryonic stem cells instructed the cells’ fate towards the foregut [44]. Also, the retinoic acid particularly specified the cell’s fate toward a posterior foregut. In this sense, due to the inhibiting role of anterior gut fate instead of posterior fate together with an ability to give rise to midgut and hindgut identities, Wnt and FGF have been used for building human intestinal organoids [45]. Creating a 3D organoid model specific for gastrointestinal epithelial cancers is challenging as it requires more tissue-specific modification for the individual niche requirements [46]. However, with the improvement of the growth condition by supplementing with Gastrin, fibroblast growth factor10 (FGF10), and p38 MAP kinase inhibitor SB202190, the R-spondin system was subsequently adapted for generating the gastrointestinal system cancer-specific organoids from Lgr5+ adult stem cells or biopsy derived tissues [4,47]. The addition of various modulators of WNT and Notch signaling pathways has also helped maintain organoid culture. Therefore, Huang et al. showed that Wnt3a, Retinoic acid, BMP as well as TGF- and Notch signaling pathway inhibitors should be added to the R-spondin culture system to activate the Wnt signaling pathway and promote the differentiation of the progenitor cells to provide a long term culture of human pluripotent stem cell- and patient-derived pancreatic cancer organoids [48]. In 2011, Sato et al. showed that similarly, the addition of Wnt-3A and nicotinamide aids in the expansion and durable culture of human small intestine and colon tissues with human colorectal cancer cells, Apc-deficient adenomas, and human metaplastic epithelia from regions of Barrett’s esophagus [49]. With a similar culture condition, van de Wetering et al. established and phenotypically annotated ‘‘paired organoids’’ derived from adjacent tumor and healthy epithelium from colorectal cancer patients [16]. In 2016, Fujii et al. improved this technique to derive a biobank of 55 colorectal tumor organoid lines and 41 matching normal colorectal organoids by collecting data from different combinations of the niche factor conditions [50].

#### 2.1.3. Niche Factors for Organoids Models to Study Liver Cancer

During early hepatogenesis, progenitor cells delaminate from the foregut endoderm to form the liver bud, and the Wnt pathway activation is crucial for the expansion of these cells [51]. Thus, human liver cancer organoids have also been successfully derived from tumor needle biopsy samples by employing the hepatocyte growth factor (HGF) in the standard R-spondin culture method without Noggin [52,53].

#### 2.1.4. Niche Factors for Organoid Models to Study Brain Cancer

The central nervous system, which is first formed as the neural plate and then as neural tube by folding and fusion, is originated from the neural ectoderm [54]. Axes are created by the co-existence of the morphogenic gradients such as the ventral-dorsal Wnt/Bmp axis and the rostral-caudal axis and the influence of factors such as retinoic acid and FGF [55]. These axes allow the epithelial tube to transform into four main regions: the forebrain, midbrain, posterior brain, and spinal cord. While the forebrain leads most of the human brain structure, including the neocortex, hippocampus, amygdala, and hypothalamus, the midbrain leads to the tectum, hindbrain cerebellum, pons, medulla, and brainstem [56]. By mimicking the endogenous patterning of the growth factors, the brain organoids could be generated through Hedgehog activation for ventral forebrain and inhibition for Purkinje neurons, Bmp4, and Wnt3a for generating granule neurons and cerebellar identities [57,58]. For glioblastoma, current in vitro models are also limited in preserving the cellular and mutational diversity of parental tumors [59]. Evidences from most of the studies over the years have shown that patient-derived glioma stem cells (GSC) are the most biologically and phenotypically related cells to the parental tumor in patients [60,61,62]. Thus Hubert et al. demonstrated that patient-derived glioblastoma organoids generated with Matrigel and exogenous EGF/bFGF had shown stem cell heterogeneity [59]. Moreover, Jacob et al. have generated a biobank of patient-derived glioblastoma organoids which have the capacity of recapitulating the cellular heterogeneity, gene expression levels, and mutational profiles of their corresponding parental tumors by using the R-spondin culture method with the same modifications of gastrointestinal organoid culture niche factors such as FGF and SB202190 [63].

#### 2.1.5. Niche Factors for Organoids Models to Study Ovarian Cancer

The simple columnar epithelial cells lining the fallopian tube in which secretory cells produce tubular fluid and ciliated cells enable the transport of the gametes, which has been implicated in the origin’s primary site of high-grade serous ovarian carcinomas [64]. For the 3D organoid model of the fallopian tube as in the intestinal tract, skin, liver, and ovary, stemness is maintained by active Wnt signaling while the R-spondin family of proteins acts as Lgr receptor agonists [65,66]. Suitably, organoids’ reproductive capacity is modulated by Wnt3A and R-spondin-1, and the Notch signaling pathway also contributes to this regulation [67]. For ovarian cancer, especially high-grade serous ovarian cancer (HGSOC), the most common type of ovarian cancer causes a 70-80% mortality rate, patient-derived 3D models and biobanks, which have stable long-term cultivation conditions, remain elusive. In 2018, Hill et al. generated a platform for functionally profiling the DNA repair in patient-derived high-grade serous ovarian cancer organoids. Their results showed that defects in replication fork protection correlated with carboplatin, CHK1, and ATR sensitivity [25]. In 2019, Kopper et al. reported the generation of organoid biobanks, which established 56 organoid lines from 32 different patients covering the spectrum of ovarian cancer, including HGSOC [26]. They derived organoids with a success rate of 65% by modification of the R-spondin culture method with an edition of small molecules such as Y-27632, ROCK (Rho-associated, coiled-coil containing protein kinase) inhibitor, A83-01, TGF-β pathway inhibitor, forskolin, and nicotinamide. And recently, Hoffman et al. established that for the stable expansion and stemness maintenance, the HGOC organoids require a niche composition with a low Wnt signaling and active BMP signaling [68].

#### 2.1.6. Synthetic Matrices for Organoids Models

As we mentioned before, although in current organoid culture methods, animal-derived hydrogels, including Matrigel and collagen, are often used as a 3D matrix component, synthetic ECM analogs are promising alternatives to native matrices. Considering that animal-derived hydrogels contain complex and variable compositions, it can be stated that they are not as amenable to controlled modifications as synthetic ones. Their risk of carrying immunogen and pathogen transfer makes them unfavorable for downstream clinical applications [69]. To overcome such challenges, the researchers designed gel-compound synthetic ECMs with adjustable molecular and physical properties that support conversion from single human tissue-derived stem/progenitor epithelial cells to organoids instead of starting with tissue fragments. The researchers functionalized the poly-ethylene glycol (PEG) hydrogel backbone with RGD (Arg-Gly-Asp), and laminin-111 that are essential for ISC expansion and organoid formation to develop well-defined matrices for the formation of intestinal stem cells and intestinal organoids. These synthetic ECMs consist of PEG-macromers modified with ECM-binding peptides and integrin-binding peptides that cross-link with matrix metalloprotease (MMP) degradation-sensitive peptides, which also support the growth and formation of endometrial epithelial organoids from single cells [70]. Moreover, it has been shown that for efficient derivation of both human and mouse hepatic organoids, yes-associated protein 1 (YAP)-mediated stiffness sensitivity and Src family of kinases (SFKs) are required since organoid growth was discovered to be highly stiffness-sensitive. For recapitulating key physical and biochemical characteristics of the hepatic microenvironment with synthetic matrices, Sorrentino et al. generated inert polyethylene glycol (PEG) hydrogels that were enzymatically crosslinked by the activated transglutaminase factor XIIIa (FXIIIa) [71]. Since aberrant matrix stiffness led to a shift in the progenitor phenotype, which results in compromised proliferative capacity, to mimic the mouse liver’s mechanical properties, PEG gels’ stiffness was tuned at physiological values (about 1.3 kPa). The soluble factors which are present in the hepatic niche such as hepatocyte growth factor (HGF) [52], the Wnt agonist R-Spondin [5,21] and fibroblast growth factor 10 (FGF10) [22]; the ECM proteins such as laminin-111, collagen IV and fibronectin have also been added to media combined with PEG hydrogel [72,73].

**Table 1:** Niche factors and ECM components utilized in the growth of cancer-specific organoids.

Cancer-Derived Organoid Type	Niche Factor		ECM Component	References
	Proteins & Growth Factors	Small Molecules		
Breast	EGF/Noggin/R-spondin Wnt-3A FGF7 FGF10	A83-01 Y-27632 SB202190 Nicotinamide	Matrigel BME * Collagen-Matrigel	[22] [35] [36]
Colon/Colorectal	EGF/Noggin/R-spondin Wnt-3A FGF10	Gastrin A83-01 Y-27632 SB202190 Nicotinamide Prostaglandin E_2_	Matrigel Growth Factor Reduced-BME *	[16] [49] [50]
Gastrointestinal	EGF/Noggin/R-spondin BMP-4	Gastrin A83-01 Y-27632 SB202190 Nicotinamide	Matrigel Growth Factor Reduced-Matrigel BME * Collagen Collagen-Matrigel PEG Hydrogel	[4] [46] [47] [70]
Glioblastoma	EGF/Noggin/R-spondin	Gastrin Nicotinamide A83-01 SB202190 Y-27632 Prostaglandin E_2_ CHIR 99021	Matrigel	[59] [63]
Liver	EGF/R-spondin Wnt-3A FGF10 HGF	Nicotinamide Gastrin Forskolin	BME * PEG Hydrogel	[52] [53] [72]
Lung	EGF/Noggin/R-spondin Wnt-3A FGF4 FGF7 FGF10	A83-01	Matrigel	[39] [40]
Ovary	EGF/Noggin/R-spondin FGF10 Heregulinβ-1 BMP-4	Nicotinamide A83-01 Y-27632 Forskolin	Growth Factor Reduced-BME * Matrigel	[25] [26] [68]
Prostate	EGF/Noggin/R-spondin Co-culture with stromal cells	EGF/Noggin/R-spondin 1 A83-01 Testosterone ROCK inhibitors	Matrigel	[21] [38]
Pancreas	EGF/Noggin/R-spondin FGF10	Wnt-3A Retinoic acid A83-01 Nicotinamide Gastrin	Matrigel GFR Matrigel Collagen	[19] [37] [48]

* Basement membrane extract.

### 2.2. Co-Cultures of Organoids

To better recapitulate the tumor or cellular microenvironment and overcome such challenges as inadequate inflammatory response due to the absence of immune, stromal, and blood cells, the co-culture system with tumor/cellular microenvironment cells is necessary [45]. The co-culture system of human primary prostate stromal cells with the human benign prostate epithelial cells increases viability and branching phenotype in human prostate organoids. In addition, this co-culture system with prostate stroma maintains differential expression of alpha-methylacyl-CoA racemase (AMACR), a well-established marker for prostate cancer, and increases the survival and passaging capacity of cancer derived-organoids [74]. When co-culturing with fibroblast cells, human renal organoids could provide a 3D model to study human renal development and disease [75,76]. Besides improving the organoid culture model’s efficiency, the main aim behind organoid co-culture models is to successfully use this system to study the interaction between cancer cells and cellular components of the tumor microenvironment. As we have discussed before, patient-derived organoid models have been proven to recapitulate many features of tumors, such as response to therapy and genetic heterogeneity. To expand the successful recapitulation feature of tumor organoids with the co-culture system, tumor microenvironment cells such as cancer-associated fibroblast and immune cells could be integrated to clarify their interaction and improve treatment strategies for a better-personalized therapy. In this frame, Ohlund et al. have developed a co-culture system with pancreatic stellate cells and mouse and patient-derived pancreatic adenocarcinoma organoids, which demonstrates the presence of two cancer-associated fibroblasts (CAFs) subpopulations into tumors: i) CAFs, which is located immediately adjacent to neoplastic cells in mouse and human pancreatic ductal adenocarcinoma tissues and have elevated expression of α-smooth muscle actin (αSMA), ii) CAFs which secreted IL-6 and additional inflammatory mediators without elevated αSMA expression [77]. In 2019, the same team further supported their study using single-cell RNA sequencing to represent the tumor microenvironment content as well as the neoplastic feature of human and mouse PDAC tumors [78]. Other than the existence of myofibroblastic and inflammatory CAFs in vivo, a new population of CAFs expressing MHC class II and CD74 but not classical co-stimulatory molecules were identified. With this study, MHC class II expressing CAFs was recognized as capable of delivering antigens to CD4+ T cells and potentially modulating the immune response in pancreatic tumors. It was also found that CAFs subtypes are representing dynamic and interconvertible states as antigen-presenting CAFs could convert into myofibroblasts upon suitable culture conditions. In addition, by using a PDAC organoid-CAF co-culture assay, Seino et al. have established that CAFs transmit a pro-tumorigenic niche signal to PDACs through the juxtacrine production of stromal Wnt ligands [79].

T cells are a vital component in cancer immunotherapy; therefore, the tumor organoid T cell co-culture is a crucial system for understanding tumor-specific T cell response and studying their interactions with tumor cells at an individual patient level. Dijkstra et al. developed a personalized patient model of a co-culture system that is able to induce the tumor-specific T cell response [80]. The authors demonstrated that co-cultures of autologous tumor organoids, which were established from mismatch repair-deficient colorectal cancer, non-small-cell lung cancer, and peripheral blood lymphocytes, can be enriched with tumor-reactive T cells from peripheral blood of the same patients.

These T cells can be used to evaluate the killing efficiency of matched tumor organoids; indeed, live imaging has shown a potent reactive T cell-mediated tumor organoids death when incubated with autologous T cells, but this effect was not seen in healthy organoids

Macrophages, especially tumor-associated macrophages (M2 Macrophages), are also subjected to TME influence. With the TME interaction, pro-inflammatory tumor-suppressive M1-type macrophages can polarize and transform tumor-promoting anti-inflammatory M2-type phenotype. Co-culturing pro-inflammatory macrophages with cancer cells cause the alteration of tumor-promoting M2 type phenotype. Neal et al. established a co-culture method based on the ALI system, which consists of minced tumor tissues resuspension in type I collagen and layered on top of pre-solidified collagen gel within an inner transwell. These systems allowed to preserve of macrophages and other native stromal populations and permitted the propagation of patient-derived organoids and tumor-infiltrating immune cells for up to 60 days [81].

### 2.3. Organoids and Viral Infections

Pathologies caused by viral infections are frequently studied with 2D cell cultures and in vivo animal models. However, the translation of these in vitro and in vivo models into clinical applications has been very disappointing as cell lines are not suitable for modeling interactions between different cell types, and many human viruses cannot effectively mimic human pathophysiology in these animal models. To better understand host-virus interactions, 3D organ-like structures, organoids, stand out as a functional model for simulating in vivo physiology and pathophysiology. After establishing brain organoid models in 2013, these models were used by Garcez et al. to test the Zika virus (ZIKV) infections during neurogenesis [82]. A flavivirus type of ZIKV, associated with decreased neuronal production, proliferative defect, and cortical progenitor cells’ death, causes malformation named microcephaly. After this study, multiple groups successfully established that the ZIKV infection targets human brain cells, which disrupts cerebral organoid cortical layers, abrogating their growth and viability as neurospheres and brain organoids [83,84]. It has been found that ZIKV treatment could also be used for the treatment of glioblastoma, an overly aggressive brain tumor with a poor prognosis. Oncolytic viruses such as ZIKA are naturally cancer-selective and suitable for genetic modifications that could increase the lytic potential and induce innate and adaptive anti-tumor immunity of the host [85]. The virus has been demonstrated to use the tyrosine kinase AXL and ligand Gas6 of the receptor for entry [86]. Chavali et al. also found that the neural RNA-binding protein Musashi-1 (MSI1) interacts with the Zika genome and enables viral replication [87]. These essential proteins associated with ZIKV infection in humans are also overexpressed in cancers, including glioblastoma multiforme. Additionally, Zhu et al. established that ZIKV was highly efficient at infecting and killing human glioma stem cells (GSC), cultured in vitro as organoids. ZIKV infection reduced GCS organoids’ cellular heterogeneity by causing loss of self-renewal and proliferation-related markers and inducing apoptosis. Infection with ZIKV-Brazil or ZIKV-Dakar slowed the glioblastoma organoid growth in the second and fourth weeks, while the form of mature organoids was seen in the third week. Thus, the researchers created a genetically modified ZIKV vaccine that still targeted brain cancer stem cells and could delay tumor growth and improve survival in organoid and animal models [88,89].

Over the last decades, there has been a rapid emergence of persistent infection with oncogenic human papillomavirus (HPV) associated with cancer in mucosal and skin tissue. Multiple studies have shown that for the HPV+ head and neck squamous cell carcinoma (HNSCC) patients, the overall and disease-free survival is significantly better than HPV- patients. However, some of the HPV+ HNSCC patients’ tumors may not respond to traditional treatments, and unusual recurrence patterns and metastasis may also occur [90,91]. Thus, Tanaka et al. developed a novel in vitro HPV+ HNSCC cancer organoid tissue lines that show many properties of the original tumors. They established the organoids by the cancer tissue-originated spheroid (CTOS) method [92]. Cancer specimens HNSCC patients were mechanically and enzymatically digested and separated into fractions, and cultured in an ultra-low culture dish. After confirming the spheroid formations, cells were transferred into Matrigel and cultured with bFGF rich media. These organoid lines show very similar histological features to original tumor tissues, as well as the equivalent expression level of cancer stem cell markers to original tumors. Moreover, the response of these organoids to cisplatin or docetaxel suggests that the HNSCC organoid model can accurately predict drug sensitivity in vivo.

The prevalence of liver diseases is increasing and induced approximately 2 million deaths per year worldwide. In addition, hepatocellular carcinoma (HCC), the most common primary liver cancer, ranks second in cancer-related deaths worldwide and is associated with hepatitis B virus (HBV) and hepatitis C virus (HCV) infections [93]. In HBV-associated HCC, transformation of the liver often causes or accompanies prolonged symptoms of chronic hepatitis, inflammation, and cirrhosis. The resulting viral load is a strong determinant of both the incidence and progression of HCC [94]. HBV also plays a vital role in regulating the accumulation and activation of cellular components of the microenvironment such as immune cells and macrophages, as well as non-cellular components of the microenvironment such as cytokines and growth factors [95]. Thus, De Crignis et al. established a patient-derived HBV infected organoid model for hepatocellular carcinoma. They showed that HBV infected patient-derived liver organoids display an early cancer gene expression signature [96]. Moreover, human liver cancer organoids derived from needle biopsies recapitulated the histology and molecular profile of primary tumors [53]. Recently, Yang et al. established hepatorganoids that prolong mice’s survival with liver failure using 3D bio-printing technology, which also allows for performing drug screening [97]. This method was further extended to generate HBV infected patient-derived models for HCC. The 3D bio-printed HCC survived well, retained the specific biomarkers of HCC, and maintained both genetic alterations and the expression profile of their original tumors [98].

Respiratory viruses, comprising severe acute respiratory syndrome coronavirus 2 (SARS-CoV-2), which causes coronavirus disease-19 (COVID-19), severe acute respiratory syndrome (SARS), and the Middle East respiratory syndrome (MERS), can cause multiple system infections in various animals. SARS-CoV-2 targets lung epithelial cells, including Alveolar type-1 cells, while influenza viruses target Alveolar type-1 and type-2 cells after in vivo mouse models infection [99,100]. In 2009, the very first influenza pandemic of the 21st century was H1N1 (H1N1pdm), which originated via multiple reassortments of swine H1N1 virus with human H3N2 virus, avian virus, and avian-like swine viruses [100]. Zhou et al. aimed to evaluate the level of infectivity of these viruses on humans by producing long-term expanding 3D human airway organoids that contain four types of airway epithelial cells consisting of ciliated, goblet, club and basal cells [101]. First, cell differentiation was achieved, which enhance the number of ciliated cells to a nearly physiological level with synchronously beating cilia in every organoid. Second, elevated levels of serine proteases were reached, which are essential for an effective infection of human influenza viruses. As a result, the H7N9/Avian-Human virus replicated more powerfully than the poorly human-infective H7N2 virus. On the other hand, the highly human-infective H1N1pdm virus replicated to a higher titer than the counterpart swan H1N1. Porotto et al. showed that a 3D lung organoid model derived from human pluripotent stem cells that include mesoderm and pulmonary endoderm and develop into branching airway and alveolar structures could be utilized for studying human parainfluenza virus 3 (HPIV3) infections [102]. As of the beginning of May 2020, we are at the 22nd week of COVID-19’s pandemic, and the number of infected people worldwide is roughly equal to the July 2009 of H1N1’s pandemic [103,104]. SARS-CoV-2 causes serious respiratory infections in humans, and the transmission of pneumonia associated with COVID-19 has not yet been truly examined. To date, only a few very recent studies have addressed SARS-CoV-2: Specifically, human bronchial and lung organoids were generated for SARS-CoV-2 research [105,106]. Besides the lung damage, SARS-CoV-2 affects several organs. Zhao et al. have shown that human liver ductal organoids are indulgent to SARS-CoV-2 infection, which impaired the cholangiocytes’ bile acid transporting functions [107]. This effect might also cause bile acid accumulation and consecutive liver damage in patients infected with SARS-CoV-2. Penninger and colleagues demonstrated that SARS-CoV-2 could directly infect human blood vessel organoids and kidney organoids, both established from human iPSCs, which can be inhibited by human recombinant soluble angiotensin-converting enzyme 2 (hrsACE2) [108]. The SARs-CoV-2 entry receptor ACE2 and transmembrane serine proteinase 2 (TMPRSS2), the receptor that the virus uses to prime the S protein (spike protein of SARS -CoV-2), are highly expressed in human airway epithelial cells [109]. Besides being a critical receptor for SARS-CoV-2, ACE2 also protects the lung from injury, which providing a molecular explanation for severe lung failure and death due to SARS-CoV infections [110]. In addition, Lamers et al., Zang et al., and Zhou et al. have demonstrated that human intestinal organoids, which were established from human adult stem cells, support SARS -CoV-2 replication [111,112]. These simultaneous studies showed that enterocytes, which are the most common cell type of the intestinal epithelium, are easily infected. Given the high levels of ACE2 expression, detection of SARS-CoV-2 RNA in anal swabs, and supporting evidence from these reports, suggested that the intestine could be a potential site of SARS-CoV-2 replication. One of the most important but not focusing aspects in COVID-19’s pandemic are the patients with cancer, which were predicted to be at increased risk of severe COVID-19 outcomes due to underlying malignancy and treatment-related immunosuppression.

The pandemic’s case series from heavily affected countries showed a high prevalence of cancer patients in the population with COVID-19 [113,114]. Especially, it has been shown that COVID-19 is indicated by poor prognosis and prolonged viral transmission in patients treated with chemotherapy, which can cause lymphocyte depletion [115]. Above the higher risk of infections for patients receiving cytotoxic chemotherapy, patients who were undergoing treatment with immune-checkpoint inhibitors have been under higher risks owing to two significant issues: the potential coincides with the COVID-related interstitial pneumonia together with uncommon immune-related lung toxicity from immune-checkpoint inhibitors; and the hypothesis that there is an association between immune-checkpoint inhibitor mechanism and COVID-19 pathogenesis in terms of cytokine-release syndrome [116]. Clinical decisions about cancer patients receiving immunotherapy in the current context of the COVID-19’s pandemic should be characterized by patient-specific evaluation, avoiding generalizations due to these extremely variable immunological statuses. Therefore, it is more urgent than ever to use microfluidic approaches to overcome the COVID-19’s pandemic due to their ability to tailor patient-specific treatment options. By creating a novel 3D complex organoid model system, researchers could reflect the interaction between the pathogen and the immune system, map the coronavirus infections, and cell-to-cell spread model together with disease interaction microenvironment [117,118]. Given the better visualization of the organoid models’ infection and heterogeneous structure, this would lead to a better understanding of viruses’ behavior in different people and be prepared for the next pandemic.

### 2.4. Advantages of the Organoid Models

Organoid models have many advantages, as they form an almost physiological model system that starts from cells and creates an middle step for in vivo transition to studying tissues and diseases: (1) The ability of organoids to remain genetically and phenotypically stable while maintaining tumor heterogeneity and being extended over a long period of time without genetic changes have accelerated the application of organoid technology, thus creating a unique opportunity to promote and consolidate basic and clinical cancer research [49,119,120,121,122,123] (2) Organoids are comparably easy to grow and can be obtained from various sources such as adult cells, ESC, IPSC, cancer cells, and primary tissues with a limiting portion of starting materials. Moreover, high quality molecular and cellular imaging techniques can be easily applied for organoids [2,52,124,125,126]. Using gene editing technologies, normal organoids can be transformed into tumor organoids that mimic genetic changes during cancer initiation and progression, as well as cells can be selected or unselected to reflect the patient’s heterogeneity and can be individually monitored [4,127]. For instance, by applying gene-editing technology, researchers have developed APC, TP53, KRAS, SMAD4 and/or PIK3CA mutated cancer driver pathway colorectal cancer (CRC) organoids, which were able to generate micrometastases when implanted into the mice. At the same time, organoids with both mutated cancer driver pathways and chromosomal instability were capable of creating large metastatic tumors when transplanted into the mice [128]. By using the patient-derived organoids, it is possible to develop biobanks and investigate diseases that are difficult to model in animals [129,130]. Recent studies have demonstrated that patient-derived tumor organoids can capture the cancer-specific genetic alterations and histopathology in individual patients that can be used for personal drug screenings, which make them suitable to correlate the genetic background of a tumor with drug response. Organoid technology has been utilized to discover cancer prognosis-related mechanisms. Broutier L et al. have shown 30 potential tumor biomarkers by systematically comparing transcriptional differences between healthy and primary liver cancer (PLC) organoid lines [17]. Among these 30 tumor biomarkers, 11 genes were reported for the first time, and 13 genes were associated with poor prognosis. (4) Organoids offer a cost-effective model for drug discovery and screening [17,131,132,133,134]. The production of cancer organoids even using simple cell culture setups makes them affordable and accessible and enables the culture to be accomplished in a short time [127] (5) Co-culturing of organoids with TME cells overcomes the inadequate inflammatory response due to the absence of immune, stromal, and blood cells and allows the study of complex interactions between TME and cancer [45]. Fujii, M. and his colleagues have demonstrated that niche factors significantly impact colorectal cancer (CRC) organoids, which can support or inhibit organoids’ growth [50].

### 2.5. Limitation of the Organoid Models

Compared to classical 2D culture models and spheroid cultures, organoids self-regulate themselves similar to *in vivo*; however, many existing organoid models are not subject to the influence of the inflammatory response as they are not supported by immune, stromal, and blood cells [119,135].

Besides, they are incapable to imitate biomechanical forces like shear stress that occurs with blood flow [136]. Due to the relatively rigid extra-cellular matrix used in organoid culture components, drug penetration may be limited. It is still difficult to culture organoids from tissues whose microenvironments and niche factors are poorly understood, like ovarian organoids. In addition to all these microenvironmental challenges, the same culture can be heterogeneous in terms of quantitative concepts such as cell viability, growth, and size due to the variability of organoid culture conditions [119,137]. Finally, in organoid models, as in many 3D models, as the organoids grow in size and volume, the core diverges from the surface and the simple diffusion process becomes insufficient to provide oxygen and nutrients. Given that the volume of waste removed from the growing cells will also become limited, only cells that come into contact with the fresh medium survive [2,138]. Overcoming all these limitations of existing organoid models, which are highly promising tools for understanding organ development, disease modeling and progression, can be accomplished by combining co-culture methodologies and microfluidic technologies that mimic in vivo conditions.

## 3. Advantages of Microfluidic-Chip Technology for Evolving Current Organoid Models

**Figure 2:**
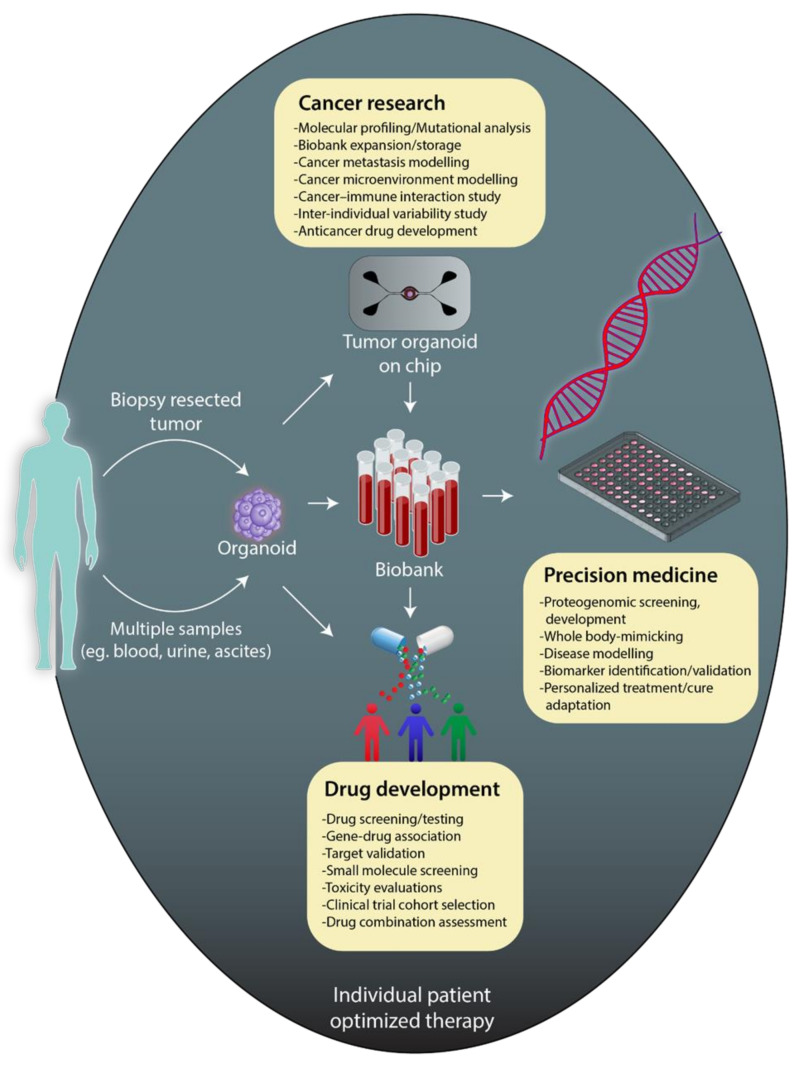
Applications of microfluidic organoid on-a-chip.

The possibility of precisely controlling many parameters in the microscale and over a thousandfold less in volume compared to the standard plate assays allowed the microfluidic technology to model physiological conditions and, therefore, the in vivo environment with a high throughput approach. The merge between microfluidics and cell biology has brought to organoids or organs-on-a-chip, i.e., “microfluidic devices lined with living cells for drug development, disease modeling, and personalized medicine” (Figure 2) [139]. Organs on-chips are miniaturized biomimetic systems that represent the key function of living humans. They usually consist of transparent 3D polymeric microchannels fulfilling the vital functions of organ structures: (a) 3D microarchitecture, which consists of multiple tissue types; (b) stimulation by dynamic mechanical and biomechanical forces; (c) functional multiple tissue integrations [140,141].

In a microfluidic organ-on-a-chip, flow conditions, nutrient supply, shear stress, input-output, and geometry can be easily controlled. Thanks to the possibility of controlling spatio-temporally the laminar flow produced through a microfluidic system, it is possible to recreate a peculiar microenvironment for developing and maintaining organoid modeling. A fluid flow can be defined as laminar when viscous forces dominate, and the flow can be described as a series of sheets that move one over the other with time-independent fluidic parameters, or turbulent when inertia forces dominate and fluid particles have a chaotic motion. In a microfluidic organ-on-a-chip, flow conditions, nutrient supply, shear stress, input-output, and geometry can be easily controlled. To design an organ-on-a-chip, it is important to identify the critical function, which needs to be reproduced to have a constructible simplified version of the real system able to model the desired function.

The organoid technology combined with the chip technology showed up for the first time in 2010 when a chip with six identical dynamic multi-micro-organoid bioreactors was used to maintain human micro organoids of liver, brain, cortex, and bone marrow each in its physiological conditions for a long culture period [142]. By using microfluidic organ-on-a-chip technology, researchers have been recently developed a variety of biomimetic organ models, such as lung [143,144], liver [145], kidney [146], intestine [147], skin [148], and heart [149]. These organo-mimetic microdevices can create micro-physiological systems that offer several advantages, such as mimicking the tissue-tissue and organ-organ interfaces to the recapitulation of a unique tissue architecture. For example, Huh et al. have shown that using a poly (dimethylsiloxane) (PDMS) microdevice containing a central microfluidics channel and two hollow side chambers simulate the structural alveolar-capillary interfaces. For these interface formations, the air overlaid human alveolar epithelial cells were cultured on one side of the porous membrane, the human lung capillary endothelial cells placed on the flowing medium on the opposite side of the same membrane to forming the alveolar-capillary barrier. The geometry of this engineered tissue interface was optimized with a thin PDMS membrane containing ECM coated pores, which stretched horizontally with cyclic vacuum suction that applied to the side hollow chamber of the cell culture channel. These induce cyclic mechanistic stretching and simulate physiological breathing movements [150]. In another study using PDMS, the microfluidic liver chip was created, which is separated by a microfabricated barrier to spare hepatocyte cells in fluid flow, mimicking the endothelial-hepatocyte interface of the liver sinusoids [151].

In microfluidic devices, precision and repeatability of mechanical automation decrease the variability compared to classical organoid cultures that require a lot of manual manipulation. Microfluidics allows us to control the space and the environment where cells have grown, and therefore cell-cell interactions, by the chip’s geometry. Moreover, it is possible to stimulate cells with mechanical [152] and/or electrical [153] stimuli. Stimulus and microfluidic nutrient supplies reduce the time required to reach maturity levels. Thus, Marsano et al. proposed a beating heart on-a-chip device, which provides a functional three-dimensional cardiac model to possibly predict signs of hypertrophic changes in cardiac phenotype by mechanical /electrical coupling and biochemical co-stimulation [154]. This can be very important in regenerative medicine and personalized medicine, where experiments on cells can influence medical decisions.

One of the key issues for organoid culture is the gradient of morphogens and cell-secreted soluble factors that in vivo arise naturally in the stem cell microenvironment. In vitro morphogens are added to induce cell-fate specification and physical segregation. In that condition, it is not possible to externally intervene in gradients, and the obtained morphogen distribution often does not reflect the normal environment and brings to different tissue patterning. Therefore, the chemical gradient in a microfluidic channel is one of the main characteristics which allows us to restore this unique heterogeneous microenvironment for organoid cultures. Due to exploiting the high control that can be obtained on laminar flows generated in microfluidic devices, it is possible to originate controlled chemical gradients. One way to produce a gradient is using a Y or a T shaped chip channels where through each inlet, a different stream is sent and meet the other at the intersection. When the two streams come into contact while keeping their laminar behavior, a gradient across the intersection is established [155]. Based on this concept, an intricate chip has been developed to create a chemical gradient perpendicularly to the flow, such as placing serpentine channels after the junction to obtain different mixing ratios to create the final gradient [156], while a cross-shaped microfluidic platform was used to generate up to two simultaneous or sequential orthogonal gradients in the central region. The cross-shaped platform was exploited to create gradients of soluble substances of interest such as morphogens in order to mimic in vivo conditions and promote stem cell differentiation, and different gradients of fetal bovine serum medium to investigate cancer cells chemotactic adaptation. Another way to create gradients in microfluidic chips is the “source and sink” method. It allows us to avoid shear stress in cells. In this method, at least two flows are employed: one solution of the desired chemicals, and the other is a solvent (usually water). The two flows circulate independently and continuously, each one in a T-shaped channel. These two T-shaped channels are connected through a chamber or a channel where gradient arises [157]. The “Source and sink” method was used to screen a diverse range of Wnt-3a and R-spondin1 concentrations for their impact on a large number of colon organoids (colonoids) [158]. With these gradient culture systems, researchers effectively determined the minimum growth factor concentrations required to elicit a physiologically-relevant colonoid phenotype. Furthermore, microfluidic flow allows to monitor shear stress that could be a problem for cells, and therefore it is possible to assure a low value of this important parameter [159,160]. On the other hand, laminar flow can also be exploited to study shear stress by using trapezoid microchannel geometries. Soffe et al., for example, studied the shear stress gradient response of human embryonic kidney cells by using trapezoid microchannel geometries [161].

Moreover, one of the significant conceptual advantages of microfluidic chip technology is controlling different factors and stimulating them within the single microfluidic device. Since it is possible to control mechanical forces such as shear, strain, and stretch forces, which affect the vascular system, [162] microfluidic chips can be used as vascular models [160], generating the flow inside these vessels that mimics biological conditions. Humans’ fluids are transported inside channels with inner diameters ranging from 3 μm of capillaries to 12 mm of aorta [161] dimensions that can be obtained in microfluidic chips. Indeed, through photolithography and soft photolithography (later explained), it is possible to produce chips with channels’ length from 500 μm to several centimeters and diameters ranging from a few micrometers to millimeters, modeling the structures of blood vessels. The possibility to reproduce the vessel is incredibly useful to replicate and study different body parts and illnesses; for example, tumor-vasculature-on-a-chip has been engineered to study nanoparticle accumulation in tumors as a result of the enhanced permeability and retention (EPR) effect [163]. Wang et al. presented a multipurpose microfluidic device that has a robust construction methodology to establish a connected, perfused vascular network from the artery to capillary beds to vein [162]. Researchers connect multiple platforms such as vasculogenesis, angiogenesis, and anastomosis by creating the capillary network inside the tissue chamber along with endothelial cell lining microfluidic channel adjacent to the tissue chamber. In this artery and vein design, angiogenesis was induced by leading the anastomosis of the microvascular network.

In a microfluidic device, it is also possible to increase both organ scale and the density at which organs can be cultured. Traditional organoid cultures suffer from limited nutrient supply and life span. During organoids’ growth, upon reaching a limiting size, their inner core can lose viability and be subject to necrosis due to the inability of oxygen, nutrients, and metabolites to reach the core through diffusion. This can be overcome with microfluidic devices where organoids are better supplied with nutrients and can be vascularized, supporting long-term cultures [163]. Nutrient supplies can be designed in order to reproduce the natural environment. Due to the fact that microfluidics provides a long-term culture system for organoids, recently, Sart et al. were able to map the structures and biological functions of mesenchymal bodies using microfluidics [164]. With microfluidic devices, nutrients, metabolites, and oxygen are delivered to organoids via laminar flow, reducing the size of the necrotic core [165] and increasing their size. The vascularization of organoids can be induced in a microfluidic chip under flow conditions helping to reduce inner core necrosis and improving organ functionality [166]. Since nutrients are brought to organoids through the flow, it is possible to control their concentration and spread in the chip by sensors, and therefore the cell density, which can be sustained higher than traditional cultures.

Finally, with improvement by microfluidic technology, organ-on-a-chip can be upgraded to barriers on-chips and body-on-a-chip models based on their function. Organs on-chips can also be used to imitate the existing physiological complexity in the human body and the interaction between different organs, so-called “body-on-a-chip.” Body-on-a-chip technology, which is made by connections of multiple organs-on-a-chip, allows the integration of multiple tissue compartments to simulate variety of organs [167]. With these applications, it is possible to study the absorption, distribution, metabolization, and elimination (ADME) of drugs throughout multiple organs [168]. In addition to create a body-on-a-chip, the most frequently measured physiological parameters in the organs-on-a-chip are mostly related to tissue barrier functions. This leads to the development of “barrier on-chips” models. Corneal [169], transepithelial [170], blood-retinal [171] and blood-brain [172] barriers on-a-chip are reported in literature. With the barrier assessment in organ-on-a-chip technology, it is possible to study both organs and barriers in an environment that reproduces their in vivo physiological functions without disrupting the cells’ viability, which is not possible in the traditional in vitro system, in turn contributing to the understanding of the unique mechanism of the diseases [173].

### 3.1. Chip Production

Microfluidics is based on chips, the core of this technology. Chips are produced with blocks of different materials (polydimethylsiloxane, silicon, glass, polycarbonate polymethylmethacrylate, polystyrene, cyclic olefin polymers, polyimide) [174] on which channels are molded or etched. In order to obtain a microfluidic chip, at least one of the dimensions of the channel must be micrometric. Besides channels, also little chambers could be present; indeed, chip patterns are designed based on the application.

**Figure 3:**
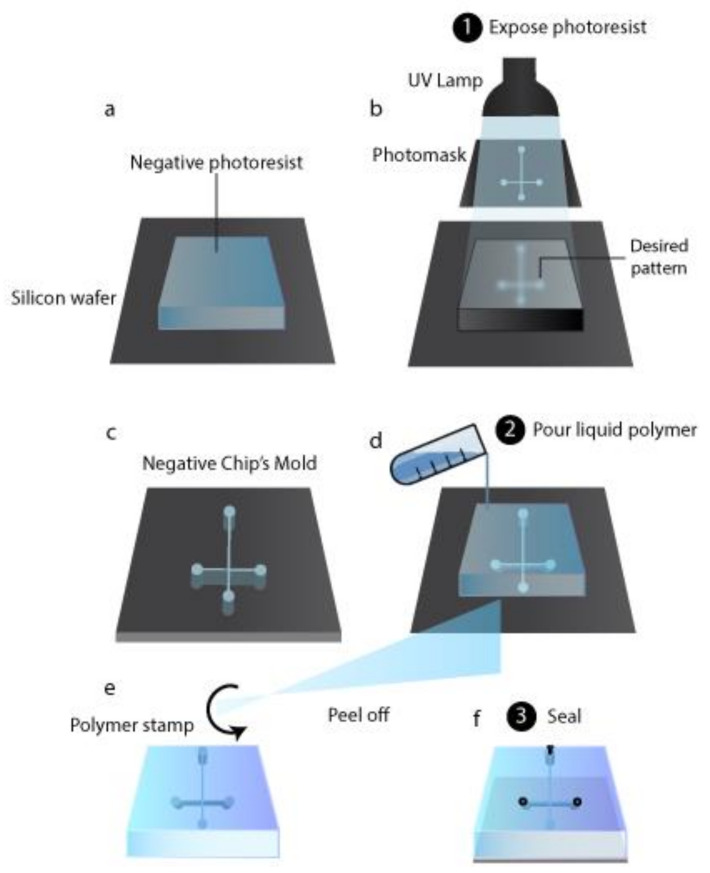
Preparation of a T-junction chip by photolithography (**a**–**c**) followed by soft lithography (**d**–**f**). (**a**) A negative photoresist is placed over a silicon wafer. (**b**) The photomask with a hole shaped on the desired chip pattern is placed over the photoresist and then illuminated. (**c**) After the development bath, only the negative photoresist exposed areas remain, obtaining the negative mold of the chip. (**d**) A liquid polymer (such as PDMS) is poured over the mold and allowed to polymerize. (**e**) The rubber-like material obtained after polymerization is peeled-off; it contains micro empty spaces, which are the channels of the chip. (**f**) The polymeric material is then sealed in its open part by bonding it to a layer, while at the opposite side, connectors are placed to allow the delivery of the fluids through channels.

Initially, chips have been produced based on production techniques used to fabricate microchips (i.e., photolithography and surface micro-matching), which named the microfluidic device. The materials utilized were glass and silicon, while nowadays, polymeric materials are preferred due to their low cost, biocompatibility, optical transparency, mechanical and chemical properties [174]. When the chip is used for cell cultures, polydimethylsiloxane (PDMS) is chosen as material since it is permeable to gas and it is characterized by a high optical transparency. On the other hand, PDMS chips suffer from channel deformation, hydrophobic recovery, leaching, sample adsorption, low resistivity to solvent, and extreme pH values. Other possible polymers are thermoplastic and hence rigid materials, which do not present PDMS drawbacks and, therefore, are preferentially used in bioanalytical microfluidic applications [175]. The photolithography technique is still used to obtain the desired pattern over a substrate. It is based on photoactive materials that form a film over a non-polymeric solid substrate such as glass or silicon. Over the photoactive material film is placed a mask shaped with the desired pattern, and the system is exposed to light, which induces a photochemical reaction. Based on the effect of photochemical reaction on photoactive materials they are categorized as negative or positive photoresistants. For a positive photoresistant, the reaction makes exposed areas soluble with a development bath, while for a negative photoresistant, the light makes it insoluble. Negative molds are the negative image of the desired structure and thus can be used in soft lithography to directly obtain chips (Figure 3a–c) [176]. Other etching techniques involve liquid etchant, a beam of ion-electron or phonon, plasma, or gases. Liquid etchants can produce an isotropic etching or etch some crystal plans in a crystalline structure preferentially. Liquid etchants allow materials to dissolve, usually through redox reactions with soluble products. Gasses can be used as etchants when they can react with a substrate and dissolve it. Beams of ions, electrons, or phonons directly remove the substrate’s atoms by collision energy [177].

In soft lithography, an etched substrate is used as a stamp to create a polymeric inverse (from negative to positive and vice versa) replication; to do that, the liquid polymer is poured on the stamp and peeled off after polymerization (Figure 3d,e) [176]. When negative molds are used as a stamp, cavities are obtained in the polymeric replication, and therefore the chip is formed. Also, positive molds can be employed; in such cases, soft lithography is followed by other microcontact print methods to produce microfluidic chips [178]. Thermoplastic polymeric chips can be obtained through thermoforming, molding them into the desired form by heating. A thermoplastic polymeric substrate, heated above its glass transition temperature, can be shaped by a stamp pressed over it, obtaining, after cooling, a glassy or semi-crystalline chip; this is called the hot embossing technique. Indeed, with the injection molding technique, pellets of the thermoplastic polymers are sent into a heated mold, which shaped them into the chip [177]. Laser ablation micromachining could also be used to produce polymeric chips. The energy breaks the polymeric bonds in the laser’s substrate, and the decomposed material is removed from the ablation region [176]. Once channels are produced to obtain the finished device, they have to be sealed in their open part by bonding a layer over the chip. The bonding procedure must not alter the channel parameters. PDMS reversibly bonds with another layer of PDMS or with glass thanks to van der Waals forces (Figure 3f). Therefore, no further fabrication procedures are required unless an irreversible bond is needed, which can be obtained through plasma treatment. For thermoplastic polymer, bonding is a critical step because it influences surface chemistry, optical transmissivity, and the microfluidic device’s geometry stability. Thermoplastic polymer chips can be bonded using an intermediate layer (indirect bonding) or without any additional materials (direct bonding). The last step is connecting the microfluidic device and the fluidic interface through connectors (Figure 3f); surface modification may be required for specific applications.

### 3.2. Flow Mechanisms

Different pumping techniques have been used to deliver fluids into microchannels and are classified as passive or active. It is important to know the differences between the possible solutions to choose the most suitable for the organoid system’s needs. Active flow mechanisms (AFMs) rely on external power sources, fields, or actuators. In biomedical microfluidics, active mechanical pumps are widely used since they allow great control over the flow. The most commonly used and commercially available mechanical pumps are syringe pumps, peristaltic pumps, and pressure/vacuum-driven pumps, whose characteristics are reported in Table 2. The modified version of these active mechanical pumps, which can also be used for multiple flow perfusions in parallel using a single device, are commercially available. The characteristics that might be useful for the organoid-on-a-chip are the possibility of controlling the pressure applied in the cell culture to avoid mechanical stress on cells and the chance to recirculate the flow and applying complex flow patterns mimicking the human body. Furthermore, by controlling both pressure and flow rate, it is possible to calculate and monitor also chip resistance and evaluate its change during the experiment. Numerous papers reported in the literature indicate the use of syringe [179,180,181,182], peristaltic [183,184], and pressure-driven pumps [185] for organoids on a chip (Table 2).

Passive flow mechanisms (PFM) do not foresee any external operator, fields, or energy supply. The examples reported in Table 3 are the principal PFMs among the numerous possible mechanisms reported in the literature. Unlike active flow mechanisms, with PFMs, it is not possible to finely adjust or quickly change a flow rate, and neither set a particular flow cycle. Furthermore, it is not always possible to generate a continuous flow, and therefore numerous papers reported in the literature focus on this issue. Since recirculation of the media is often necessary for organoid-on-a-chip, the possibility to achieve it is reported in Table 3. PFMs are widely studied since they allow the developing of tubing free, space-saving devices, which can be placed into incubators, and creating the necessary microenvironment for tissues or cell cultures. However, some PFMs still depend on external actuators, such as the rocking platform used for continuous gravity-driven flow, which might preclude the possibility of placing the device in the incubator (depending on platform size). It is possible to use multiple devices simultaneously, and therefore grow multiple cultures. This is possible as a result of the easiness of these devices; for example, in literature is reported the production of two lines of 8-device arrays of two-well hanging droplet device all in a single chip [190].

A rocking platform has been used to create a continuous dynamic flow of media that mimics blood recirculation in a liver organoid-on-a-chip [191]. Human midbrain organoids were maturated on a chip containing three interconnected chambers of which the central one is dedicated for the organoids while the lateral ones are filled with different volumes of medium in order to generate an hydrostatic pressure and therefore the flow inside the chip allowing the active transport of the nutrients, and maintaining low the shear forces [159]. Surface tension-driven flow can be used to obtain the flow in an open microfluidic device, which differ from the traditional devices since not only the inlet and outlet wells are open to the air but also at a minimum one other area of the chip [190]. The upside of the open microfluidic is the easiness of cells or tissue displacement besides the minimal disturbance, particularly useful in shear sensitive cultures.

**Table 3:** Comparison among passive mechanical pumps.

PFM	Actuation	Recirculation
**Gravity-driven flow**	Using a rocking platform which allows the fluid to flow inside interconnected microchannels continuously [191] -Placing the fluid reservoir in a higher position than the chip [192]	Using the rocking platform, the medium continuously recirculate in the chip [191]
**Hydrostatic pressure-driven flow**	-Connecting liquid column (reservoirs) to the inlet and outlet of the chip, it can be controlled via their differential heights	Recirculation can be achieved by: -manually refilling the inlet reservoir with the medium collected at the outlet [193] -siphon-based auto-filled hydrostatic pressure-driven passive micropump [194]
**Surface tension-driven flow**	-Placing over the inlet port of a microchannel a drop with a smaller radius than the one placed at the outlet port [195]	It is not possible to recirculate the medium
**Osmosis-driven flow**	Using a membrane permeable only to solvent; the flow is obtained thanks to different concentrations across that layer [196]	It is not possible to recirculate the medium

### 3.3. Monitoring Cell Parameters

It is essential to check cell parameters and therefore indagate their metabolism, growth, and keep track of changes in the culture. Biomolecules and oxygen concentrations, their gradients as well as the pH, influence the fate of tissues and cells, and therefore their control is crucial to generate and maintain 3D cell cultures. In particular, it is essential to monitor culture parameters when extended culture time is required, such as for drug testing studies, as well as to indagate the dynamic response to the pharmaceutical compounds tested. Microfluidic organoids on-a-chip are not suitable for traditional analytical methods, which require large sample volumes and manual sampling that often causes system disturbance. Sensors have been frequently used since they need a low volume of sample, can work on-line and transmit results very fast, are non-invasive, and have a small size allowing their in situ placement without perturbing the system.

**Table 4:** Overview of sensors that can be used to monitor cell parameters in microfluidic chips.

Parameter	Sensor	Functioning	Peculiarities
pH	Ion Sensitive Field-Effect Transistor (ISFET)	pH is determined through the current flowing between the terminals called source and drain. Indeed, the device’s potential is established in the solution using a detecting interface (specifically sensitive to H+) and a reference electrode (called gate potential).	Miniature size, durability, rapid response, and ion selectivity [197].
	Light addressable potentiometric sensor (LAPS)	LED or laser light is used to generate photocurrent, which intensity depends upon ion concentration in the analyzed solution affecting the potential establish between a detecting interface (specifically sensitive to H+) and the reference electrode.	Can be used as imaging devices allowing to visualize the ion concentration as a two-dimensional map [198]
	Potentiometric sensor	pH is determined from the voltage measured between a reference electrode and a sensitive electrode obtained exploiting metal oxides sensitivity to H+	To make a space-saving device, screen printing has been used to produce thick film based potentiometric pH sensors by depositing on the same substrate both the reference and the sensitive electrodes [199].
	Optical measure	pH is determined by measuring the absorbance of the culture medium containing pH indicator phenol red. The absorbance can be determined using a transmitted-light microscope and a digital camera with a CCD sensor. The generated light by a microscope lamp passes through (in-plane) the microfluidic device and reaches the CCD sensor where its intensity is measured, and therefore absorbance is calculated [200].	
Oxygen concentration	Amperometric sensor	Oxygen is determined using cathode and anode electrodes placed in the electrolyte solution. Indeed, the current generated by oxygen reduction at the cathode depends on its partial pressure. From oxygen partial pressure the gas concentration in the solution is determined since these two values are directly proportional. As a cathode electrode, it is possible to use a noble metal dipped into the medium, i.e., a direct amperometric sensor or a Clark’s type sensor in which a gas-permeable membrane separates the sensor from the electrolyte.	To improve the miniaturization of the amperometric sensor, it is possible to measure oxygen concentration through local pH changes due to oxygen reduction at the cathode with a pH-sensitive ISFET [201].
	Optical sensor	Oxygen concentration is measured dependently on the quenching of a dye fluorescence exerted by molecular oxygen. Dye molecules are excited by a particular electromagnetic radiation and emit light to return to the normal energy state. When oxygen is present in the sample, the emitted light is limited or altered for its interaction with the dye. The measured effects (fluorescence amplitude, phase shift, or fluorescence lifetime) are inversely proportional to the oxygen partial pressure.	The dye can be incorporated in a polymer to create a sensor site [202] or in a membrane coating the entire surface of the cell culture vessel to monitor spatial change in oxygen concentration [203]. Dye excitation is pursued by using LED, and the signal is registered with a photodetector. These three elements are placed together in commercially available sensor devices. However, it is preferable to separate the optical set up from the cell environment to avoid sterilization. Also, an inverted microscope can be used to read sensor spots.
Metabolites	Functionalized sensors	Metabolites can be measured with electrochemical sensors based on enzymes able to react with these molecules. The enzyme can be immobilized in a membrane or directly on the electrode surface. By-products of the enzyme reactions are reduced or oxidized at a polarized electrode, and the reaction is electrochemically followed. Electrodes can also be functionalized with antibodies obtaining immunobiosensors sensitive to specific analytes.	

The sensors reported in Table 4 are the main devices which can be employed to monitor critical parameters in the cell environment. One of the parameters that needs to be monitored to control the cell microenvironment is the pH, which is influenced by cell activity. Indeed, lactate and/or CO2 produced by cell respiration causes the acidification of the culture medium to a particular extent due to the reduced volume of the cell chamber in microfluidic devices. Ion Sensitive Field-Effect Transistor (ISFET) was used in medical applications and microfluidics [204,205]. Using different detecting interfaces with ISFET, it is also possible to analyze other biologically important ions such as Na+, K+, Ca2+, Cl-, simple molecules, e.g., glucose, and more complex biochemical compounds like DNA, several antibodies, neurotransmitters, and enzymes [197]. Due to ISFET characteristics, these devices have been used to produce multiparametric sensor chips to monitor the cell microenvironment [206,207]. As for ISFET, also for Light addressable potentiometric sensor (LAPS) by changing the sensitivity of the sensitive layer, it is possible to measure different analytes such as Ca2+, Li+, SO42- and NO3- and urea and also to create a multisensory LAPS by using different sensitive materials on different sites of the sensing surface [208]. The easy encapsulation and high sensitivity of LAPS make these devices ideal for being integrated into microfluidic systems in order to monitor cell parameters. Indeed, these sensors have been used in microfluidic systems: LAPS integrated with a PDMS chamber have been used for real-time pH monitoring of the medium where hepatoma HepG2 cells were cultured and treated with doxorubicin in order to assess the extracellular acidification rate [209]; and, in a microphysiometer for pH monitoring to track the metabolism of human breast cancer cells [210]. Furthermore, by cultivating adherent CHO-K1 on the LAPS surface, it was possible to image the extracellular acidification [198]. The cell’s environment’s pH can be controlled through potentiometric measures by placing the electrodes in a microfluidic device [211]. To assess the anaerobic or aerobic pathways in cell metabolism and determine the respiration rate, it is essential to measure oxygen concentration. Oxygen concentration in microfluidic devices can be measured electrochemically using a cathode and an anode electrode placed in the electrolyte solution [212]. Furthermore, optical oxygen sensors have been integrated into microfluidic devices avoiding oxygen consumption and the production of toxic byproducts during the measurement [213,214]. To indagate cell metabolism, it is necessary to assess metabolites such as pyruvate, glucose, and lactate; in order to measure their concentration, functionalized electrodes can be used.

In literature, many works are describing the multiparameter monitoring of organoids on a chip. A multisensory-integrated organoid-on-chips platform is reported, which allows an automated, in situ, and continual monitoring of organoids [214]. The platform has been used to study the drug-induced toxicity of two model systems; heart and liver on a chip and liver-cancer-heart on a chip. To monitor different organoid biomarkers, a microfluidic immunobiosensor chip has been used. It was realized a microelectrode system with 500 nm working, counter and reference electrodes. Owing to the microfluidic platform’s design, working electrode-related operation steps, i.e., functionalization, detection, and regeneration, are fully automated and take place in the sensing chip. It is connected to the organoid module but is equipped with valves and channels, which allow us to isolate the solutions involved in the electrode operations from the cells, avoiding any toxic effects. Physical parameters are as well monitored. Temperature is controlled through a temperature probe, and the pH is tested with an optical sensor, which measures the absorbance of phenol red. An optical sensor based on ruthenium dye fluorescence was used to monitor oxygen concentration. Furthermore, the observation of organoids can be performed through miniaturized microscopes. It has also been reported the development of a microfluidic platform with electrodes integrated into the channels surrounding the organoid compartments to monitor oxygen and lactate for the studying of tumoroids obtained from patient-derived breast cancer stem cells [215]. These sensors are made up of platinum, which is deposited in the channels and then modified by hydrogel deposition. Oxygen sensors are modified with a hydrogel layer to limit the diffusion, while a hydrogel containing lactate oxidase is used for lactate sensors. The lactate reaction catalyzed by the enzyme produces as a by-product hydrogen peroxide, which is electrochemically active, giving information about the analyte concentration. The counter electrode (naked platinum) and reference electrodes (Ag/AgCl deposited over the electrode) are as well preset in the channels.

### 3.4. Bioprinted Organs-on-a-Chip

Organ-on-a-chip models were designed to recapitulate the physiological, microenvironmental, and structural functions of human organs and for multiple tumor types by using bioprinting. Owing to its ability to contain various materials, cell lines, and biochemical factors, 3D bioprinting may represent precise control over spatial heterogeneity in the TME for a variety of cell types and predefined biobanks in a reproducible manner [216]. Bioink is the soft biomaterial that has the ability to printed together with living cells. Naturally derived hydrogels are the most commonly used biomaterials for 3D bioprinting technologies [217]. Alginate and gelatin are also widely used bioink materials due to their mild crosslinking conditions and biocompatibility capability [217]. For instance, Grolman, J.M., et al. have demonstrated a system with breast adenocarcinoma (MDA-MB-231) and macrophage (RAW 264.7) cell lines that were printed in the hydrogels. Printed hydrogels have been modeled in distinct spatial distributions and variable geometrical shapes by extrusion-based 3D bioprinting technique [218]. The results showed that geometric cues regulated the paracrine signaling network and further regulated the breast cancer metastatic process by tumor intravasation into the bloodstream. Moreover, Skardal et al. have developed a functional perfusion-driven microfluidic multi-tissue organ-on-a-chip system for liver, heart, and lung organoids [219]. Individual tissue constructs could also be combined with serial fluid-flow in modular microreactor systems formed by conventional PDMS soft lithography and molding. This system also allows the integration of additional tissues. Another example of 3D bioprinting is the HeLa cells mixed with the hydrogel to study the epithelial-to-mesenchymal transition (EMT) process [220]. The HeLa/hydrogel composed of gelatin, alginate, and Matrigel was fabricated by forced extrusion in a layer-by-layer method and further forged into cell-biomaterial constructs grid shape. Aggregated HeLa cells started to transform into fibroblast-like spindle morphologies, an indication of EMT. Overall, the integration of the organoid-on-a-chip platform with the bioprinting technology presents a promising direction for highly efficient, automated, and integrated functional tissue models that will serve as an alternative to animal and human models.

## 4. Patient-Derived Organoids to Improve Cancer Therapeutics

The requirement for personalization of cancer therapies results from the tumor heterogeneity due to every cancer’s unique genetic nature. Even though the overlaps in the driver gene activities of tumors from different patients, the various mutational landscape between patients mostly leads to different therapeutic outcomes for treatments with the same drugs and cause the failure of “one-size-fits-all” approaches of conventional medicine, in particular for cancer [221]. Conventional drug and biomarker screenings directly on patient materials or patient-derived models for personalized treatment options are currently limited from the number of cells due to the small amount of starting materials and costs due to low throughput. In order to increase the feasibility of personalized therapeutic options, the development of technologies that enables sensitive detection of cancer “signals” at the same time of disease evolution with high throughput will be of great interest. Hence, microfluidics is a technology that handles fluids at the microscale with high accuracy; it provides a solution for managing the small samples such as patient-derived tumor cells, solid tumors, or biopsy materials and the functional and genetic analysis of these small heterogeneous cell populations with an increased throughput. It also provides a cost-effective high level of automation and allows the set-up of complex models for cancer studies. Microfluidic technology provided a physiologically similar context to recapitulate the complex process of cancer metastasis and extravasation. As a result, integration of bioengineering with microfluidics, researchers utilize a 3D ECM-inspired hydrogel system, which can be integrated with a microfluidic delivery system [222]. This system has been used as a metastasis-on-a-chip model for organoids that consists of multiple chambers, hosting liver, lung, and endothelial cell populations. The fluorescently labeled colorectal cancer organoids hosted in the interconnected main chamber can enter circulation and reach other organoid sites such as the liver and lung. This metastasis-on-a-chip model mimics tumor spreading under controlled conditions [223]. By using a similar system, Mazzocchi et al. demonstrated that patient-derived mesothelioma tumor organoids efficiently recapitulate the mutational landscape of the tumor and used for identified targetable mutation, which they probe from the effects of an experimental drug compound [184]. As another example, Hao et al. established a microfluidic bone-on-a-chip for spontaneous growth of a 3D, mineralized, collagenous bone tissue, which is used to study breast cancer metastasis to bone marrow. Based on the principle of simultaneous growth dialysis, the model contains two compartments for osteoblastic tissue growth and normal culture medium flow [224]. This design mimics the breast cancer bone colonization’s unique hallmarks with an extended-term growth for up to 1 month. In another research, a multi-organ microfluidic bone chip was used to mimic lung cancer metastasis to the brain, bone, and liver [225]. These multi-organs-on-a-chip organoids were divided into different chambers, including upstream “lung” and three downstream “distant organs,” with three PDMS layers and two thin PDMS microporous membranes that bonded each other to form three parallel microchannels. For mimicking the blood circulation, the culture medium flow-through while a circulation vacuum was applied to reproduce breathing movement. With this physiologically relevant lung cancer metastasis system, cancer cells formed a tumor mass that showed epithelial-mesenchymal transition and invasive capacity.

By combining these advanced engineering approaches, which range from gene sequencing to tumor organoid engineering with organs-on-a-chip technology, could allow the screening of cancer immunotherapies by recreating the intrinsic and extrinsic properties of a TME. Immunotherapy’s efficacy could depend on patient-specific mutations and the expression of specific biomarkers within the immune microenvironment, making predictive screening of immunotherapy options crucial at a single patient’s level [226]. Thus, Aung et al. developed a perfusable multicellular breast cancer tumor-on-a-chip platform utilizing different cell populations such as cancer cells, monocytes, and endothelial cells within a gelatin hydrogel. The ability of the endothelial cells in the outer layer to migrate towards the chemokine gradient produced by the serum-containing circulatory medium was used to create an endothelial layer around the bilayer hydrogel structures. This platform examined the effect of cancer cell-monocyte interaction on T-cell recruitment, where the T-cells were dispersed within the perfused media and allowed to infiltrate. The addition of monocytes to the cancer cells improved T-cell recruitment, and T-cell recruitment differences have been associated with chemokine secretion [227]. Using the microfluidics technology, modeling a 3D tumor tissue that exhibits microenvironmental heterogeneity is possible as invaluable cancer models for a wide range of clinical issues. As an example, Hassel et al. demonstrated an orthotopic organ chip model of non-small-cell lung cancer (NSCLC) to recapitulate tumor growth, dormancy in lung airway, and the therapeutic response to tyrosine kinase inhibitors (TKIs) that associated with breathing motions [228].

Besides, the successful application of microfluidic technology for the maintenance and expansion of different types of patient-derived tumor cells, or liquid biopsies for personalized drug screening and the diffusion kinetics of drugs is another important issue. In vitro and in vivo drug testing sometimes bring different results as a consequence of issues related to drug distribution in vitro. Microfluidic technology allows precise control of drug distribution, increases the cellular fidelity of organoids in a limited time, and improves the dose-response accuracy. Recently, Schuster et al. have developed an automated high-throughput microfluidic platform that enables culturing tumor organoids for the continuous monitoring of 3D growth and biochemical analyses [229]. The platform consists of a 3D culture with a 200-well array chamber and an overlayed multiplexer fluid control device. Using this organoid culturing platform, the researchers could use a dynamic chemotherapy regimen in parallel with several standard chemotherapy utilized to pancreatic ductal adenocarcinoma patients in the clinic. This dynamic drug screening platform allows for testing combination chemotherapy by delivering of pulse of drugs to the desired array of tumor organoids. In another study, Herland et al. created fluidically coupled vascularized organ on-chips, which can predict pharmacokinetic parameters for orally administered nicotine and intravenously injected cisplatin [230]. Gut, liver, and kidney chips were utilized to study nicotine pharmacokinetics, while using coated bone marrow, liver, and kidney chips to study cisplatin pharmacodynamics. The chips were connected by sequential robotic liquid transfers of a blood substitute through their endothelium-lined channels. The prediction of pharmacodynamics matches the patient data previously reported. Another microfluidic platform has been developed to expand and identify the small-cell lung cancer patients derived organoid drug responses under physiologically relevant flow conditions [231]. This flow system allowed stable nutrient and oxygen provision, while a continuous flow of drug-containing medium was delivered. A first-line drug sensitivity test was performed to evaluate the treatment response using this system, and it was shown that, surprisingly, cells in the small-cell lung cancer patients derived organoid core regions survive. In contrast, cell death occurs in the outer regions. Similar to this study, Shirure et al. have been designed a perfused microvascular network for tumor cells and patient-derived organoids that also mimic the flow and dimensions of the in vivo communication between the developing tumor and the arterial end of the capillary [232]. Thus, quantitative in vitro-to-in vivo translation of pharmacokinetics and pharmacodynamics parameters and the estimation of drug absorption, distribution, metabolism, and toxicity through fluidically coupled and bound organ on-chips has great potential in the development of the design of drug administration regimens for phase-I clinical trials.

In the light of all these approaches that we have summarized, it is possible to have a new era in personalized treatment approaches by using microfluidic systems that provide controlled environments for circulating tumor cell tracking, genetic screening, biomarker analysis, phenotypic drug susceptibility tests, and disease modeling to adapt and develop patient-specific treatments.

## 5. Possible Limitations of Current Organoids on-a-Chip Technologies

Despite the huge potential of organoids-on-a-chip, it is still challenging to standardize and scale-up the current organoid-on-a-chip models. Most of the approaches require external pumps, tubing, and connectors to operate, reducing the accessibility and increasing the systems’ cost. All these steps also reduce the reproducibility of the microfluidics-organoid-on-chip models. Moreover, it can take almost a month to produce organoids derived from patient biopsies or stem cells, which is too long for clinicians to decide on personalized therapy options. More advanced engineering and screening efforts such as bioprinting are needed for especially the ADME approach of drugs throughout multiple organs.

## 6. Conclusions and Future Perspective

Microfluidics organoid on-a-chip is an almost-physiological technology that imitates organ development and human diseases, and it holds great potential in therapy response-prediction, drug development, and personalized medicine. Over the past decade, various microfluidic approaches have been established to recapitulate physiological microenvironments, which heavily rely on mimicking the cell to cell interactions. One of the extensive power of organs-on-chips is that they provide a way to carry out the experiment scale from a cellular level to a tissue and finally at the organ level. This technology allows us to begin by studying the effect of a single microenvironment component such as immune cells or external effectors like drugs and monitoring them with a combination of multiple microenvironment components together with numerous mechanical cues. As an integral part of the tumor’s ecosystems, tumor microenvironment modeling is a cutting-edge field in cancer research, and taking this field on a micro-scale with microfluidics organs on-a-chip has excellent potential to control and observe a variety of cellular response. While monitoring the cellular response and numerous mechanical cues and modulate them based on the desired condition, it is also possible to visualize all in real-time at high resolution. It is necessary to underline that microfabrication is a reductionist way of the human body within multiple modules of organoids on-chips, and one of the main advantages of this technology is the recapitulation of inter and intra-patient complexity in a controlled system. Using these micro-scale systems, it is easy to integrate data obtained from organ-on-a-chip models with animal tissues and the result obtained from animal models prior to testing it with human tissues for personalized medicine.

Further, such systems’ dynamic-personalized nature also relies on the ability to combine the regarding read-outs, which is collecting from person-derived blood samples, urine samples, primary tissue, cells, and stem cells. Together this is known as precision medicine, which provides a person-specific assessment of drug efficacy as well as personalized strategies for disease progression. Although subsequent microfluidic setups will need to address current technical limitations, the multi-organs on-a-chip so-called body on-a-chip model, which may provide an experimentally tractable human system, holds the infinite potential for personalized medicine and bring basic research closer to a clinical practice.

## Figures and Tables

**Table 2:** Comparison among active mechanical pumps.

Features	Peristaltic Pump [186,187]	Syringe Pump [186,187,188]	Pressure Driven Pump [186,189]
**Flow Rate Range**	2 μL/min–10 L/min	0.012 nL/min–0.3 L/min	nL/min–mL/min
**Working characteristics**	Periodically compression of a flexible tubing	Controlled motion of the syringe piston	Pressure is applied to a sealed reservoir pushing liquid through tubes
**Flow characteristics**	-Pulsatile flow -Low stability -Flow recirculation is possible -Large volumes can be dispensed -Only the mean flow rate can be controlled	-Constant flow -Different stability levels according to device quality -Only a fixed volume of liquid can be dispensed	-Complex flow patterns can be performed -High stability -Flow recirculation is possible -Both pressure and flow rate can be controlled
